# SMR Peptide Modulates Tumor-Derived Extracellular Vesicles microRNA and Inflammatory Transcript Signatures in TNBC

**DOI:** 10.3390/cells15060550

**Published:** 2026-03-19

**Authors:** Ming-Bo Huang, Fengxia Yan, Uswa Jadoon, Jennifer Y. Wu, Dara Brena, Erica L. Johnson, Jonathan Stiles, Lily Yang, Brian M. Rivers, Vincent C. Bond

**Affiliations:** 1 Department of Microbiology, Biochemistry, and Immunology, Morehouse School of Medicine, Atlanta, GA 30310, USA; ujadoon@msm.edu (U.J.);; 2 Department of Community Health and Preventive Medicine, Morehouse School of Medicine, Atlanta, GA 30310, USA; 3 School of International and Public Affairs, Columbia University, New York, NY 10027, USA; 4 Winship Cancer Institute, Emory University School of Medicine, Atlanta, GA 30322, USA; 5 Cancer Health Equity Institute, Morehouse School of Medicine, Atlanta, GA 30310, USA

**Keywords:** apoptosis-associated speck-like protein (ASC), caspase-1, exosomes, extracellular vesicles (EVs), inflammasome, microRNA (miRNA), NLRP3, SMR peptide, TNBC, tumor-derived EVs (tEVs)

## Abstract

**Highlights:**

- What changes with SMRwt? Extracellular vesicle microRNAs shift to tumor suppression.
- What signaling is affected? Inflammasome cytokine transcripts are reduced in cells.

**Abstract:**

Triple-negative breast cancer (TNBC) is an aggressive subtype lacking targeted therapies and characterized by pronounced heterogeneity and widespread dysregulation of microRNAs (miRNAs) that influence epithelial-to-mesenchymal transition (EMT) and metastasis. Tumor-derived extracellular vesicles (tEVs) further contribute to TNBC progression by transporting oncogenic cargo that can enhance pro-inflammatory signaling. The synthetic SMRwt peptide has been suggested to modulate oncogenic pathways; however, its effects on EV miRNA composition and inflammatory transcript profiles in TNBC remain unclear. Here, we investigated whether SMRwt alters tEV-associated miRNAs and cytokine transcript signatures relevant to EMT and inflammasome-linked pathways. Extracellular vesicles were isolated from SMR-treated and untreated MDA-MB-231 cells, followed by nanoparticle tracking analysis and small RNA sequencing. SMRwt treatment enriched 11 tumor-suppressive miRNAs (including Let-7a-5p, Let-7b-5p, miR-24-3p, miR-26b-5p, miR-92a-3p, miR-93-5p, and miR-496) previously associated with the regulation of proliferation, EMT, migration, and metastasis. We also observed modest, non-significant decreases (1.01–1.27-fold) in oncogenic miR-1200, miR-374a-5p, and miR-937-3p, which have been implicated in the progression of breast, lung, and bone malignancies. Complementary transcriptomic profiling using the NanoString nCounter Breast Cancer 360 Gene Expression Panel (NanoString Technologies, Inc., Seattle, CA, USA) demonstrated reduced expression of inflammasome-associated cytokines in TNBC cells relative to non-tumorigenic controls, including a log_2_ fold change of −1.15 for IL 1β (MDA-MB-231 vs. MCF10A). These transcript-level changes suggest potential modulation. Additionally, SMRwt suppresses ASC-mediated caspase-1 activation and reduces IL-1β secretion, thereby inhibiting NLRP3 inflammasome signaling. Therefore, we infer that SMRwt simultaneously restores tumor-suppressive miRNA networks and suppresses inflammasome-driven inflammation, supporting its potential as a dual-target therapeutic strategy for TNBC.

## 1. Introduction

Breast cancer (BC) remains one of the most prevalent and deadly malignancies among women worldwide, with metastasis as the leading cause of mortality, and the molecular mechanisms driving tumor progression and dissemination are not fully understood. Recent evidence underscores the importance of intercellular communication [1,2,3,4,5,6,7,8,9] and inflammatory signaling in shaping the tumor microenvironment and promoting metastasis. These include the interplay between EVs, inflammasome activation, and epithelial–mesenchymal transition (EMT) as a critical axis in breast cancer pathogenesis [1,2,3,4,5,9,10,11,12,13]. 

EVs (30–150 nm) transported by cancer cells—tumor-derived EVs (tEVs)—carry proteins, lipids, DNA, and diverse RNAs, enabling them to remodel the tumor microenvironment. tEVs promote tumor growth, survival, angiogenesis, immune suppression, and pre-metastatic niche formation by transferring oncogenic cargo, including miRNAs and signaling molecules. They also propagate inflammatory mediators that activate EMT-related pathways, contributing to invasion, metastasis, and therapeutic resistance.

The inflammasome, particularly NLRP3, is a key innate immune sensor whose activation generates IL-1β and IL-18. In BC, dysregulated inflammasome signaling supports tumor initiation and metastasis. EVs can transport inflammasome activators, amplifying inflammation and priming epithelial cells toward EMT [2,3].

MicroRNAs (miRNAs) are small non-coding RNAs that regulate gene expression post-transcriptionally. Normal EVs are known to selectively package and deliver miRNAs to recipient cells, thereby modulating gene expression in a paracrine or systemic manner. Alternatively, in breast cancer, tEV miRNAs have been implicated in promoting EMT, enhancing stemness, and conferring resistance to therapy. For instance, miR-21 and members of the miR-200 family are frequently enriched in tumor-derived EVs and have been shown to regulate EMT-related pathways. The ability of EVs to reprogram recipient cells through miRNA delivery adds another layer of complexity to the tumor-promoting functions of these vesicles [4].

Together, EV-mediated signaling, inflammasome activation, and EMT form a synergistic mechanism underlying BC aggressiveness. Understanding this network may reveal opportunities to disrupt tumor-promoting communication and improve clinical outcomes.

Chronic inflammation also contributes to BC development. Cytokines derived from tumor-infiltrating lymphocytes (IL-2, TNF-α, IFN-γ, IL-17, IL-21, and IL-22) and adipocytes (IL-1β, IL-6, TNF-α, MCP-1, CCL2, CXCL8, CXCL1, and CXCL10) shape the inflammatory microenvironment, whereas adiponectin and other anti-inflammatory mediators counterbalance these effects [14,15,16,17]. Because adipose tissue constitutes up to 56% of breast volume, adipocyte-derived cytokines significantly influence local inflammation [18]. Additional factors such as the breast tissue microbiome, genomic instability, and DNA damage further contribute to inflammatory responses associated with BC risk and progression [19].

Triple-negative breast cancer (TNBC) is an aggressive subtype of breast cancer lacking ER, PR, and HER2 expression, resulting in limited therapeutic options and poor clinical outcomes. Increasing evidence indicates that TNBC progression is driven not only by tumor-intrinsic signaling pathways but also by dysregulated microRNAs (miRNAs) [20,21,22,23,24,25,26,27,28], heightened inflammatory activity, and extracellular vesicle (EV)-mediated communication. In particular, activation of the NLRP3 inflammasome promotes IL-1β maturation and secretion, supporting tumor growth, immune evasion, EMT, and metastatic spread. Because miRNA dysregulation, inflammasome activation, and EV cargo alterations are interconnected processes, therapeutic strategies that simultaneously target these pathways may offer a multipronged approach to controlling TNBC progression.

Within this context, the secretion modification region (SMR) peptide has emerged as a promising bioactive molecule with the potential to modulate these tumor-promoting mechanisms. Our group and others have previously demonstrated that SMRwt can regulate oncogenic signaling at the protein level and alter cellular stress and survival pathways in multiple cancer models [29,30,31,32,33]. SMR peptides display anti-inflammatory properties, including inhibition of inflammasome activation and cytokine release, and can interfere with EV-mediated communication and EMT, thereby reducing cellular invasiveness and metastatic potential. 

In addition to these protein-level effects, SMR-derived exosome miRNAs exhibit high stability and strong regulatory capacity, enabling them to influence proliferation, apoptosis, angiogenesis, and immune modulation [34,35]. Compared with other EV-associated RNAs, SMR-associated miRNAs offer several advantages: (1) they remain stable in various tissues and body fluids, often protected by protein complexes that prevent degradation; and (2) they efficiently participate in intercellular regulatory activities, modulating the behavior of target cells through anti-apoptotic, pro-proliferative, angiogenic, and immunomodulatory effects [34]. Thus, the role of SMR-associated EV miRNAs in TNBC biology warrants further mechanistic investigation. 

Despite these promising findings, the capacity of SMRwt to regulate miRNA expression, exosome biogenesis, and inflammasome activity has not been previously evaluated. To address this gap, the present study investigates how SMRwt influences the exosome–inflammasome–EMT axis in TNBC cells. Specifically, we examine whether SMRwt alters exosome release and cargo composition, suppresses NLRP3 inflammasome activation, modulates EMT progression, and reprograms miRNA-mediated regulatory networks. By defining these interconnected effects, this work aims to establish a mechanistic foundation for the development of SMR-based therapeutic strategies that simultaneously target inflammatory signaling and oncogenic miRNA pathways in TNBC.

## 2. Materials and Methods

### 2.1. Study Design and Experimental Overview

**Figure 1:**
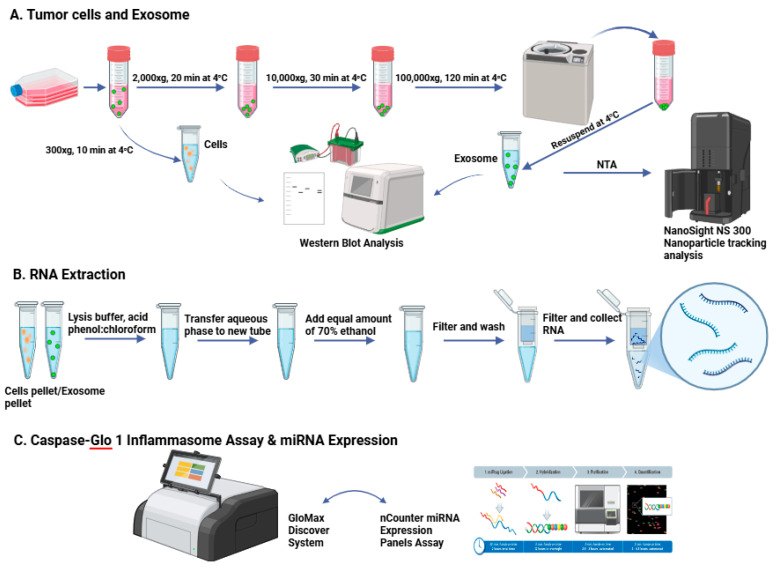
Experimental procedure. (**A**) TNBC cell culture and exosome isolation. TNBC cell lines were cultured under standard conditions, and EVs were isolated from conditioned media using ultracentrifugation at 100,000× *g* for 120 min at 4 °C. EV pellets were resuspended in PBS and characterized by nanoparticle tracking analysis (NanoSight NS300, Malvern Panalytical Inc. Wesborough, MA, USA) and Western blotting for EV markers. (**B**) RNA extraction and quality assessment. Total RNA was extracted from both cells and EVs using an miRNeasy Mini Kit (Qiagen Inc., Germantown, MA, USA). The protocol involved lysis, phenol–chloroform extraction, ethanol precipitation, and column purification. RNA concentration and purity were measured using a NanoDrop™ spectrophotometer, and integrity was assessed with an Agilent Bioanalyzer (Agilent Technologies, Santa Clara, CA, USA). (**C**) Inflammasome activation and miRNA expression analysis. Inflammasome activation was quantified using the Caspase-Glo® 1 Inflammasome Assay (Promega, Madison, WI, USA) according to the manufacturer’s instructions. Luminescence was measured using the GloMax® Discover System. For microRNA profiling, RNA samples were analyzed using nCounter® miRNA Expression Panels (NanoString Technologies, Seattle, WA, USA) to quantify miRNA expression levels. All assays were conducted in triplicate under optimized conditions to ensure reproducibility. Created with BioRender.com.

This study investigated the role of the SMR peptide in modulating EV-mediated inflammasome activation and microRNA (miRNA) signaling in TNBC. The experiment integrated cell-culture-based peptide treatment and EV isolation and characterization. EVs were isolated from conditioned media using a differential ultracentrifugation workflow adapted from our previously published methods [5]. Briefly, culture supernatants were sequentially centrifuged at: 300× *g* for 10 min to remove cells, 2000× *g* for 20 min to remove debris, and 10,000× *g* for 30 min to remove large vesicles and apoptotic bodies. The clarified supernatant was then filtered through a 0.22 μm PES membrane and ultracentrifuged at 100,000× *g* for 70 min at 4 °C using a Beckman Coulter Type 50.2 Ti fixed-angle rotor (k-factor = 69). Pellets were washed once in sterile PBS and subjected to a second 100,000× *g* spin for 120 min for purification. The final EV pellet was resuspended in PBS and stored at −80 °C. EV concentration and size distribution were assessed by NTA using a NanoSight instrument under standard settings. Samples were diluted to 1 × 10^8^–1 × 10^9^ particles/mL and recorded in triplicate. EV protein markers were analyzed by immunoblot. EV-enriched markers CD63 and ALIX were detected. To contextualize EV-associated miRNAs with EMT pathways, cellular EMT protein markers were analyzed following our previously published approach, including the epithelial marker E-cadherin and mesenchymal markers Vimentin [5,9,10,36]. RNA extraction, inflammasome activity measurement, and miRNA expression profiling were performed, followed by bioinformatics pathway and network analysis (Figure 1).

### 2.2. Cell Culture and SMR Peptide Treatment 

MDA-MB-231 human TNBC cells were cultured under standard culture conditions (37 °C, 5% CO_2_) in complete growth medium appropriate for the specific TNBC cell line used (e.g., RPMI supplemented with 10% FBS and 1% penicillin–streptomycin). For all experiments involving EV isolation, EV quantification, EV uptake assays, and cell treatments with EVs or SMR peptide, culture media were prepared using exosome-depleted FBS (System Biosciences, Palo Alto, CA, USA, SBI; Exo FBS™, Cat# Exo-FBSHI-250A-1). This commercial EV-depleted FBS is processed by large-volume ultracentrifugation and filtration to remove bovine EVs according to the manufacturer’s protocol. For experiments involving SMR peptide treatment with 10% exosome-depleted FBS, cells were seeded at equal densities and exposed for 24 h to either wild-type SMR peptide (SMRwt) or untreated EV. Untreated (UT) cultures served as controls. Conditioned media were collected for EV isolation, and parallel cell cultures were harvested for RNA extraction and inflammasome assays.

### 2.3. EV Isolation and Characterization

EVs were isolated from conditioned media using a different ultracentrifugation method. Briefly, media were sequentially centrifuged at 4 °C, 300× *g* (10 min) to remove cells and 2000× *g* (20 min) to remove dead cells and large debris and 10,000× *g* (30 min) to remove larger vesicles, followed by ultracentrifugation at 100,000× *g* for 120 min to pellet EVs. The EV pellets were washed in cold PBS and centrifuged again at 100,000× *g* for 90 min before resuspension in PBS for downstream analyses. EV size and purity were assessed using nanoparticle tracking analysis (NTA) with a NanoSight NS300 instrument. Data are reported as mean ± standard deviation.

### 2.4. RNA Extraction and miRNA Expression Profiling

Total RNA, including small RNA species, was extracted from both whole-cell pellets and purified EVs using an miRNeasy Mini Kit (Qiagen) according to the manufacturer’s protocol. RNA concentration and purity were evaluated by a NanoDrop™ spectrophotometer, and RNA integrity was assessed using an Agilent 2100 Bioanalyzer small RNA chip. miRNA expression profiling was performed using the nCounter® Human miRNA Expression Panel (798 miRNAs). Raw counts were normalized using nSolver Analysis Software v4.0 downstream differential expression analyses. Differentially expressed miRNA common between SMRwt and UT was compared using a two-sample independent test, and the significance thresholds were defined as: * *p* < 0.05, ** *p* < 0.01, *** *p* < 0.001, and **** *p* < 0.0001. These analyses were conducted using GraphPad Prism, version 10. 

### 2.5. Inflammasome Activation Assay

**Figure 2:**
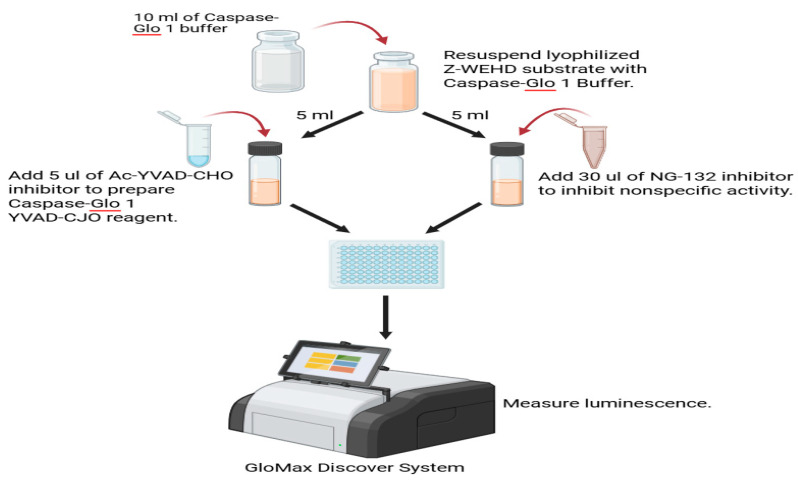
Schematic diagram of the Caspase-Glo® 1 Inflammasome Assay cell-based protocols. The Caspase-Glo® 1 Inflammasome Assay is designed for use with multiwell plates in 96-well formats, making the assay ideal for automated high-throughput screening of caspase activity or inflammasome activation. Cell washing, medium removal, and multiple pipetting steps are not required. This novel caspase-1 assay system enables a more efficient and effective assessment of inflammasome activation, allowing for high-throughput screening of inflammasome modulators. Created with BioRender.com.

Inflammasome activation was quantified using the Caspase-Glo® 1 Inflammasome Assay (Promega) following the manufacturer’s instructions. Briefly, cells were seeded into white 96-well plates at equal density and treated with SMR peptide or left untreated. After treatment, the cells were incubated with Caspase-Glo 1 reagent, which contains a luminogenic caspase-1 substrate. Luminescence, proportional to active caspase-1 levels, was measured using a GloMax® Discover System and normalized to background controls. All assays were performed in biological triplicate. Statistical comparisons between treatment groups were conducted using one-way ANOVA followed by Tukey’s multiple-comparison post hoc test in GraphPad Prism v10.0 (Figure 2).

### 2.6. Differential miRNA Expression and Bioinformatic Analyses

Differential miRNA expression between UT and SMRwt conditions was assessed using DESeq2 with Benjamini–Hochberg false discovery rate (FDR) correction. miRNAs with |log_2_ fold change| ≥ 1 and adjusted *p* < 0.05 were considered significant. Hierarchical clustering and heatmap visualization were performed for 27 upregulated miRNAs using the pheatmap R package, applying Euclidean distance and average linkage. Expression values were row-scaled to emphasize relative differences across samples. Venn diagrams comparing 40 top-ranked miRNAs were generated using the VennDiagram package in R. Validated and high-confidence predicted miRNA–mRNA interactions were retrieved from miRTarBase, miRecords and TarBase using the multiMiR package in R. An miRNA-–pathway enrichment matrix (−log10 adjusted *p*-values) was constructed in R. Pathways with no significant enrichment across miRNAs were removed. The top 20 miRNAs and top 20 pathways were selected based on the number of non-zero enrichment values to prioritize highly connected features. The matrix was row-scaled (z-score) to emphasize relative enrichment patterns and visualized using hierarchical clustering with the pheatmap package. miRNAs with at least one enriched pathway were retained for downstream analyses. Kyoto Encyclopedia of Genes and Genomes (KEGG) pathway enrichment analysis was performed for the 11 selected miRNAs, using the top 10 pathways per miRNA, with FDR correction, using the clusterProfiler package. Pathways with adjusted *p* < 0.05 and ≥5 target genes were considered significant. An integrated miRNA–mRNA interaction network highlighting key regulatory hubs was constructed and visualized using Gephi 0.10.1. KEGG pathway enrichment dot plots were generated using ggplot2, with pathway ordering facilitated by the tidytext package. All R scripts used for data processing and visualization are available from the authors upon request.

## 3. Results

### 3.1. Venn Diagram of the Top 40 Differentially Expressed miRNAs in Untreated (UT) and SMR-Peptide-Treated Wild-Type (WT) TNBC Cells

**Figure 3:**
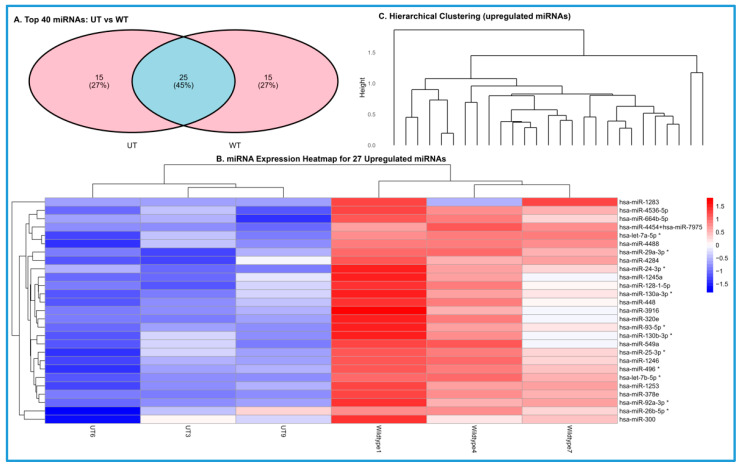
Venn diagram of the top 40 differentially expressed miRNAs in UT and SMRwt groups. (**A**) The Venn diagram illustrates the distribution and overlaps of the top 40 most differentially expressed miRNAs identified in untreated (UT) and SMR-treated WT TNBC cells. Of these, 25 miRNAs (45%) are shared between both groups, while 15 miRNAs (27%) are unique to UT and 15 miRNAs (27%) are unique to SMR-WT. This comparison highlights both shared and condition-specific miRNA signatures, suggesting that SMR treatment induces a partially distinct miRNA regulatory profile. (**B**) The lower panel displays the corresponding heatmap of normalized miRNA expression levels. Red denotes higher expression and blue denotes lower expression relative to mean-centered values. SMR treatment resulted in robust upregulation of multiple tumor-suppressive and EMT-inhibitory miRNAs, including hsa-let-7b-5p, hsa-miR-34a, and members of the miR-200 family. These miRNAs are known regulators of pathways such as PI3K/AKT, Wnt/β-catenin, and key EMT transcriptional programs. Asterisks (*) indicate the most highly enriched miRNAs within the dataset. (**C**) The upper panel shows hierarchical clustering of 27 significantly upregulated miRNAs in SMR-treated WT TNBC cells relative to UT controls. The dendrogram structure demonstrates clear segregation of SMR-treated samples from UT samples, indicating that SMR peptide induces a coordinated shift in miRNA expression.

Analysis of the top 40 differentially expressed miRNAs revealed that 25 miRNAs were shared between the UT and SMR-treated WT groups (Figure 3). In contrast, 15 miRNAs were unique to the UT condition, and 15 were unique to the SMR-treated WT group. These profiles indicate that SMR treatment is associated with a distinct subset of miRNA expression changes compared with untreated TNBC cells. The distribution of shared and unique miRNAs is summarized in the Venn diagram shown in Figure 3A (image showing: “UT: 15”, “Overlap: 25”, “WT: 15”).

### 3.2. SMR Peptide Remodels the miRNA Landscape and Segregates Treatment Groups by Hierarchical Clustering 

Hierarchical clustering and heatmap analysis identified 27 miRNAs that were significantly upregulated in SMR-treated TNBC samples compared with the untreated (UT) group (Figure 3B, lower panel). The heatmap illustrates the expression intensity of these miRNAs, with red indicating higher and blue indicating lower expression values. The upregulated set includes has-let 7b-5p, miR-34a, and several miR-200 family members, each of which has been implicated in suppressing oncogenic signaling, promoting differentiation, and inhibiting metastatic plasticity in TNBC. Several of the most enriched miRNAs (marked with *) showed >1.5-fold increases following SMR exposure, highlighting the magnitude of SMR-driven transcriptional reprogramming.

The upper panel of Figure 3C shows the hierarchical clustering dendrogram generated from these 27 miRNAs. The clustering pattern clearly separates SMR-treated samples from UT controls, indicating that SMR exposure results in a distinct miRNA expression profile. Replicates within each treatment group cluster tightly together, supporting the reproducibility of the observed expression patterns. The dendrogram structure reflects coordinated expression changes across multiple loci rather than isolated differences at individual miRNAs. Together, these analyses demonstrate that SMR treatment is associated with a consistent and statistically significant shift in miRNA expression across TNBC samples.

### 3.3. SMR-Responsive miRNAs Converge on Oncogenic and Stress-Regulatory Pathways Relevant to TNBC Biology

**Figure 4:**
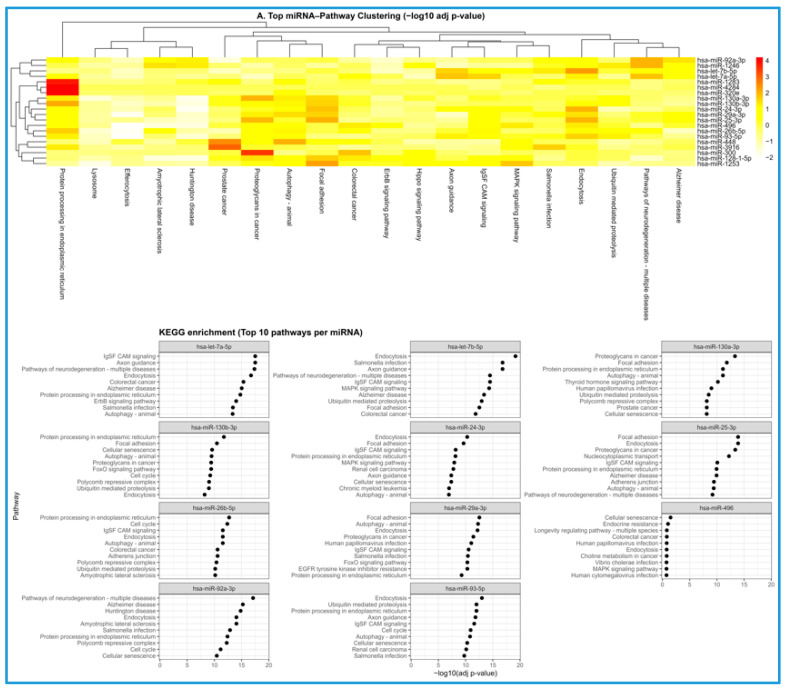
(**A**) Heatmap of differentially enriched miRNA–pathway associations in breast cancer. The heatmap illustrates hierarchical clustering of the top enriched KEGG pathways (*x*-axis) and differentially expressed BC-associated miRNAs (*y*-axis), visualized using −log10 adjusted p-values. Warmer colors denote stronger enrichment significance. Distinct clustering patterns reveal groups of miRNAs that contrast common biological pathways, identifying coordinated regulatory modules. Several miRNAs—including hsa-miR-92a-3p, hsa-miR-128-1-5p, hsa-miR-25-3p, and members of the hsa-miR-130 family—exhibit strong associations with pathways known to promote breast cancer progression. Highly enriched pathways include ErbB signaling, MAPK signaling, ubiquitin-mediated proteolysis, protein processing in the endoplasmic reticulum, focal adhesion, and pathways implicated in cancer, apoptosis, endocytosis, and neurodegenerative disease. These enriched clusters suggest that breast cancer-related miRNAs co-modulate interconnected oncogenic processes essential for tumor growth, invasion, metabolic adaptation, and stress survival. (**B**) KEGG pathway enrichment analysis for miRNAs differentially expressed between WT and UT conditions. This panel depicts the top 10 KEGG pathways enriched for each differentially expressed miRNA between SMR-treated WT and UT groups. Pathways are ranked by their adjusted p-values (represented as −log10(adj. *p*-value) on the *x*-axis). Several miRNAs show convergent enrichment patterns across cancer-relevant and stress-response pathways, including endoplasmic reticulum protein processing, MAPK signaling, axon guidance, and cellular senescence, suggesting shared regulatory effects. Notably, hsa-miR-130b-3p and hsa-miR-25-3p are highly enriched for focal adhesion and proteoglycans in cancer, pathways associated with enhanced invasion and metastatic potential. Members of the let-7 family (let-7a-5p and let-7b-5p) are strongly linked to neurodegeneration-associated signaling and MAPK pathway regulation, consistent with their roles in repressing proliferation and supporting differentiation programs. The enrichment of cellular senescence pathways in hsa-miR-496 and hsa-miR-93-5p suggests regulatory effects on stress-induced growth arrest mechanisms. Collectively, these enrichment signatures highlight the functional impact of SMR-responsive miRNA expression changes on biological pathways that differentiate WT from UT conditions.

To investigate the functional significance of the differentially expressed miRNAs identified in SMR-treated TNBC cells, KEGG pathway enrichment analyses were performed. Hierarchical clustering of the top miRNA–pathway associations (Figure 4A) revealed coordinated enrichment patterns across multiple miRNAs. Several BC-associated miRNAs demonstrated strong interactions with pathways known to promote tumor progression, including ErbB and MAPK signaling, ubiquitin-mediated proteolysis, and focal adhesion, as well as global cancer pathways. The presence of distinct clustering groups underscores the cooperative nature of miRNA-based regulatory networks, where multiple miRNAs converge on shared oncogenic processes.

Pathway-level evaluation for individual differentially expressed miRNAs (Figure 4B) further highlighted convergence on regulatory nodes central to TNBC stress adaptation and survival. miRNAs such as hsa-miR-130b-3p, hsa-miR-25-3p, and hsa-miR-92a-3p enriched strongly for pathways driving cell motility, ECM remodeling, and tumor–stroma interactions, while let-7 family members aligned with MAPK and neurodegeneration-associated modules commonly linked to differentiation and proliferative control. Enrichment of pathways related to protein folding, ER stress, endocytosis, and cellular senescence suggests that SMR-responsive miRNAs may also influence proteostasis mechanisms and the cellular stress environment. Together, these patterns reveal that SMR-induced miRNA changes target a diverse yet interconnected set of pathways involved in proliferation, migration, stress handling, and cell fate determination.

### 3.4. miRNA–Pathway Interaction Network Reveals Central Regulatory Hubs in SMR-Treated TNBC Cells

**Figure 5:**
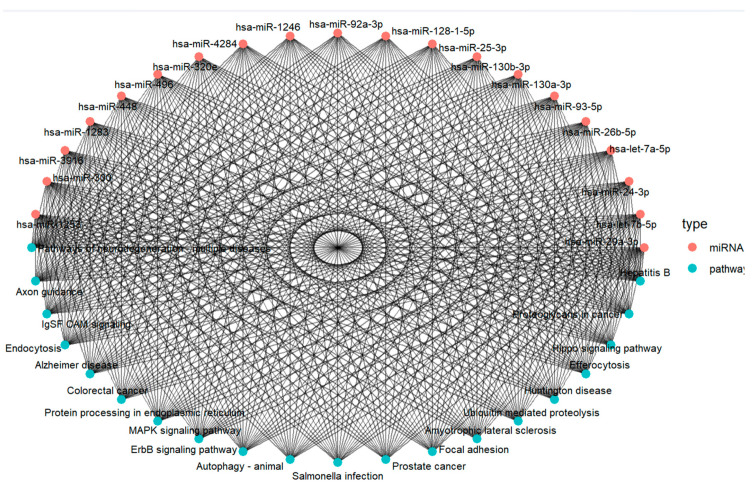
Integrated miRNA–pathway interaction network in breast cancer. The network diagram illustrates the interaction landscape between differentially expressed BC–associated miRNAs (red nodes) and their enriched KEGG pathways (blue nodes). miRNA–pathway mapping and pathway enrichment analyses reveal extensive connectivity, indicating that individual miRNAs regulate multiple cancer-relevant pathways, while several pathways are jointly influenced by numerous miRNAs. Key oncogenic and tumor-suppressive pathways enriched in the dataset include ErbB signaling, MAPK signaling, focal adhesion, ubiquitin-mediated proteolysis, endocytosis, autophagy, and colorectal/prostate cancer signaling modules. The high degree of cross-pathway regulation suggests coordinated miRNA-driven modulation of proliferative, metastatic, and survival mechanisms in BC. The dense interconnectivity underscores the potential cooperative regulatory effects of miRNA clusters in shaping the TNBC molecular phenotype and highlights candidate pathways for targeted therapeutic intervention.

The miRNA–pathway interaction network generated from the predicted targets of SMR-associated miRNAs is shown in Figure 5. The network consists of miRNAs (red nodes) and KEGG pathways (blue nodes), connected by edges representing predicted regulatory associations.

Analysis of network topology demonstrated that a subset of miRNAs—miR 29a-3p, miR-24-3p, let-7a-5p, miR-30a-3p, and miR-92a 3p—exhibited the highest degree values, indicating that these miRNAs had the largest number of pathway connections. These high degree nodes represent the most extensively connected miRNAs in the dataset.

The network also displayed a modular organization, with visually identifiable groups of miRNAs sharing overlapping pathway associations. For example, miR-29a-3p and miR-24-3p clustered together, sharing multiple pathway connections. Members of the let-7 family formed another cluster with shared interactions. These clusters reflect regions of dense connectivity within the network. Overall, the interaction map reveals a highly interconnected structure in which a limited number of miRNAs contribute disproportionately to total pathway associations.

### 3.5. Integrated microRNA–Gene Interaction Network Reveals Central Regulatory Hubs in SMR-Treated TNBC Cells

**Figure 6:**
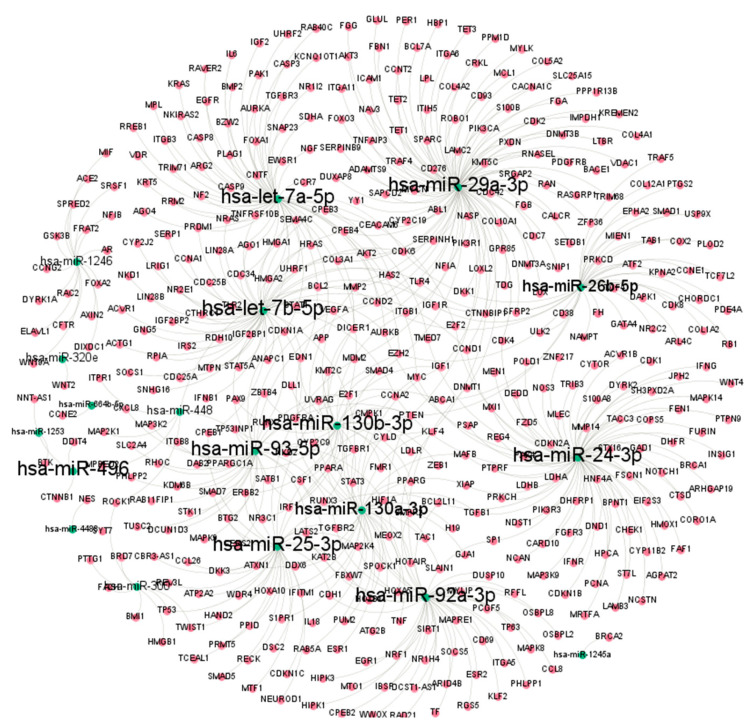
Predicted microRNA–gene interaction network in TNBC cells. The network displays predicted interactions between significantly altered microRNAs (green nodes) and their corresponding target mRNAs (red nodes). The highly interactive miRNAs hsa-let-7a-5p, hsa-miR-29a-3p, hsa-miR-26b-5p, hsa-miR-130b-3p, hsa-miR-92a-3p, and others occupy central regulatory positions. Edges represent validated or high-confidence predicted target relationships aggregated from standard microRNA target databases. The dense clustering indicates extensive post-transcriptional regulation, with several miRNAs converging on shared mRNA targets involved in pathways relevant to TNBC biology, including proliferation, apoptosis, and cytoskeletal remodeling. This network highlights key microRNAs that may mediate the transcriptional response to SMR peptide treatment.

An integrated microRNA–mRNA interaction network was constructed using differentially expressed microRNAs and their predicted or experimentally validated gene targets. The global network topology is shown in Figure 6, where microRNAs and mRNA targets are represented as nodes connected by regulatory edges.

Several microRNAs exhibited high degree centrality within the network. The most connected nodes included has-miR-29a-3p, has-miR-24 3p, has-miR-26b-5p, has-miR-93-5p, has-miR-25-3p, and let-7 family members (let-7a-5p and let-7b-5p). These microRNAs were associated with the largest sets of SMR-responsive mRNA targets.

The network also displayed distinct clusters of interconnected mRNA nodes. The mRNA targets were enriched for genes annotated to biological processes such as extracellular matrix organization, apoptosis regulation, immune-related signaling, and RNA processing. These clusters formed localized neighborhoods of densely connected genes. Overall, the integrated network reveals a highly interconnected regulatory architecture, with a subset of microRNAs contributing disproportionately to the total number of observed microRNA–gene interactions.

### 3.6. SMR Peptide Treatment Upregulates Tumor-Suppressive and Regulatory miRNAs in MDA-MB-231 Cells

**Figure 7:**
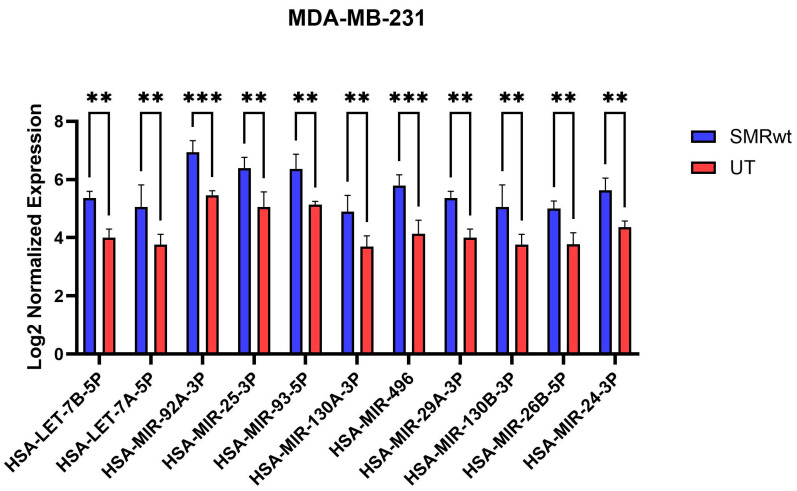
Levels of differentially expressed miRNA common between SMRwt and UT on MDA-MB-231 breast cancer cells. Bar graph showing the log2-normalized expression levels of miRNAs that were differentially expressed and shared between SMRwt-treated and UT MDA-MB-231 cells. Blue bars represent expression levels under SMRwt treatment, while red bars represent untreated controls. Error bars indicate mean ± SEM. Asterisks denote statistical significance between SMRwt and UT for each miRNA (** *p* < 0.001, *** *p* < 0.0001). The panel highlights consistent upregulation of multiple miRNAs under SMRwt treatment compared to the UT condition.

Quantitative expression analysis revealed that SMRwt treatment significantly increased the levels of all tested miRNAs in MDA MB 231 cells compared with untreated (UT) controls (Figure 7). The upregulated miRNAs included has-let 7b-5p, has-let-7a-5p, has-miR-92a-3p, has-miR-25-3p, has-miR-93-5p, has-miR-130a-3p, has-miR-496, has-miR-29a-3p, has-miR-130b-3p, has-miR-26b-5p, and has-miR-24-3p.For each miRNA, SMRwt treatment produced a statistically significant increase in log2 normalized expression values (*p* < 0.01–0.001), and the direction of change was consistent across all measured replicates. The magnitude and uniformity of these increases indicate that SMRwt induces a coordinated upregulation of multiple miRNAs in MDA MB 231 TNBC cells. No miRNAs in the panel showed a decrease relative to UT controls.

### 3.7. SMRwt-Derived EVs Suppress Extracellular Caspase-1 Activity in a Cholesterol-Dependent Manner

**Figure 8:**
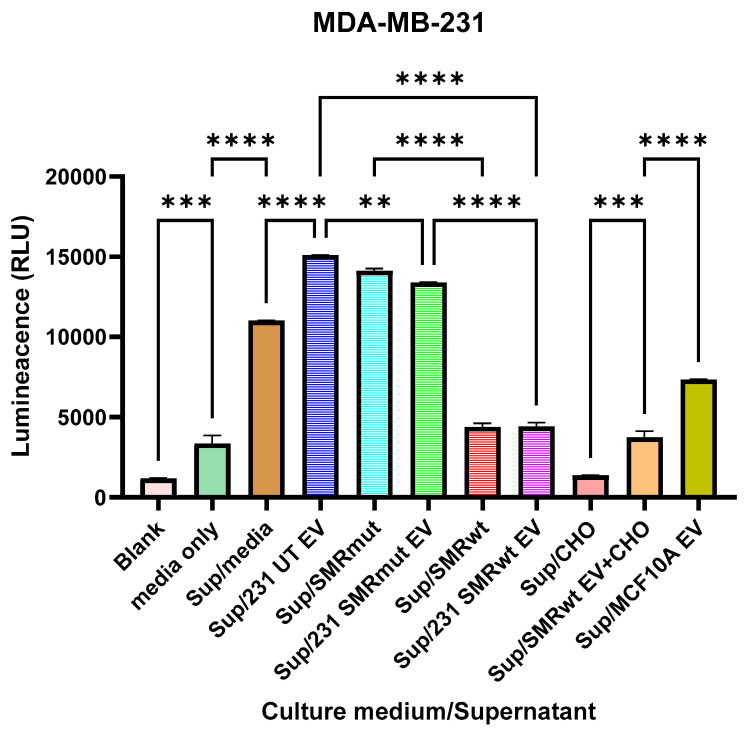
Caspase-Glo®1 Inflammasome Assay for released caspase-1 in culture medium. MDA-MB-231 BC cells were cultured in RPMI-1640 medium supplemented with 10% exosome-depleted FBS and treated for 24 h with one of four specific EV preparations: (1) EVs from untreated MDA MB 231 cells (231UT EV), (2) EVs from SMRmut-treated cells (231 SMRmut EV), (3) EVs from SMRwt-treated cells (231 SMRwt EV), or (4) SMRwt EVs isolated from SMRwt-treated cells whose membrane cholesterol was experimentally restored (SMRwt EV + CHO). For comparison, EVs from nonmalignant MCF10A cells were also evaluated. After treatment, 50 µL of clarified culture supernatant from each condition was transferred to a white 96-well assay plate. An equal volume of either Caspase Glo® 1 Reagent or Caspase Glo® 1 YVAD CHO Inhibitor Reagent was added, and luminescence (RLU) was recorded using a GloMax® Multi+ Microplate Detection System. The background signal from blank and media-only controls was minimal. EVs derived from 231UT and 231 SMRmut cultures induced high caspase 1 activity, consistent with inflammasome activation. In contrast, 231 SMRwt EVs significantly suppressed caspase 1 release. Notably, cholesterol-restored SMRwt EVs (SMRwt EV + CHO) partially rescued the SMRwt-mediated inhibitory phenotype, supporting a role for EV membrane cholesterol content in regulating EV-dependent inflammasome signaling. This is consistent with reports that lipid composition—especially plasma membrane and EV cholesterol—modulates vesicle uptake and downstream inflammatory signaling [37,38]. EVs from MCF10A cells induced lower caspase 1 activity relative to MDA MB 231 EVs. Data are means ± SEM. Statistical significance determined by one-way ANOVA with appropriate post hoc testing; ** *p* < 0.005, *** *p* < 0.001, **** *p* < 0.0001.

Extracellular vesicles (EVs) were isolated from the conditions below MDA MB 231:

(1) untreated cells (231 UT EVs), (2) cells expressing the SMR mutant peptide (231 SMRmut EVs), (3) cells expressing the wild-type SMR peptide (231 SMRwt EVs), and (4) SMRwt-expressing cells cultured under cholesterol-depleted conditions (SMRwt + CHO depletion EVs). EV uptake by naïve MDA MB 231 cells was confirmed, and recipient cells were assessed for IL1β expression as well as extracellular caspase 1 activity. Exposure to 231 SMRwt EVs produced significantly lower IL1β expression compared with cells treated with 231 UT EVs or 231 SMRmut EVs. In contrast, cholesterol-depleted SMRwt EVs did not increase IL1β levels, showing values comparable to those observed with untreated controls. Extracellular caspase 1 activity measurements revealed significant suppression of caspase 1 luminescence in cultures treated with 231 SMRwt EVs relative to UT and SMRmut EV conditions (Figure 8). Cholesterol-depleted SMRwt EVs displayed restored caspase 1 activity, indicating that the reduction in caspase 1 observed with SMRwt EV treatment was not present under cholesterol-depleted conditions. EVs derived from the nonmalignant MCF10A cell line exhibited caspase 1 activity levels lower than UT and SMRmut EVs. Overall, these data show that SMRwt-derived EVs alter IL1B and caspase 1 readouts in MDA MB 231 recipient cells and that cholesterol depletion of EV-producing cells modifies these responses.

## 4. Discussion

### 4.1. SMRwt Remodels the miRNA Landscape in TNBC Cells 

Our integrated experimental and computational analyses indicate that SMRwt treatment restructures the miRNA profile of MDA-MB-231 TNBC cells in a selective and functionally coherent manner. Rather than broadly altering global miRNA expression, SMRwt induces a defined subset of miRNAs that were absent or minimally expressed in untreated cells. These SMR-responsive miRNAs cluster around pathways known to regulate epithelial identity, cytoskeletal organization, and migratory behavior—patterns consistent with the EMT-suppressive phenotype observed in downstream assays.

The overlap analysis of the top 40 regulated miRNAs shows that untreated and SMR-treated cells share a basal miRNA core, reflecting common TNBC housekeeping programs. Importantly, each condition also possessed a distinct miRNA subset. The SMR-induced set appears enriched for tumor-suppressive or epithelial-restoring regulators, whereas the untreated-specific miRNAs included elements commonly linked to invasive or EMT-promoting networks in aggressive TNBC. This shift does not simply amplify or silence existing miRNAs but instead suggests a coordinated remodeling of the regulatory landscape, introducing new miRNA circuits while dampening pro-invasive ones.

Collectively, these findings support a model in which SMRwt exerts its anti-migratory and EMT-modulating effects, in part through targeted miRNA reprogramming. By promoting miRNAs associated with epithelial maintenance and suppressing those linked to TNBC aggressiveness, SMRwt appears to push the cells toward a more stabilized, less motile phenotype. This miRNA-level remodeling provides a mechanistic framework that aligns with the pathway and network alterations demonstrated in subsequent analyses and highlights exosome-associated miRNA regulation as a central component of SMRwt’s biological activity (Figure 3, Figure 4, Figure 5, Figure 6 and Figure 7).

### 4.2. SMR Peptide Reprograms an miRNA Network That Suppresses EMT and Oncogenic Signaling in TNBC

The Figure 4 heatmap showed 27 miRNAs significantly upregulated in SMR-peptide-treated TNBC cells compared with untreated (UT) controls, with consistent red intensities across biological replicates in SMR-treated samples and lower expression in UT. Hierarchical clustering (Figure 4, lower panel) cleanly separates UT and SMR-treated groups, indicating a coordinated and biologically meaningful shift in miRNA expression. Several co-regulated clusters align with functional modules implicated in PI3K/AKT, Wnt/β catenin, cell cycle control, and EMT suppression, suggesting interring miRNA coordination. These patterns echo pathway-level results (Figure 5 and Figure 6), supporting a model in which SMR peptide remodels not only individual miRNAs but an entire regulatory network that governs TNBC aggressiveness.

The 27 miRNAs upregulated in SMR-treated TNBC cells include several species previously associated with tumor-suppressive or epithelial stabilizing functions. For example, miR 200 family members are well known for repressing epithelial–mesenchymal transition (EMT) by targeting the transcriptional repressors ZEB1 and ZEB2. Likewise, miR 34a has been established as a p53 regulated tumor suppressor with roles in controlling cell cycle progression and apoptosis. Let 7 family miRNAs have been reported to antagonize oncogenic RAS signaling and contribute to differentiation and growth restraint [36]. The coordinated upregulation of these miRNAs in SMR-treated samples suggests that the peptide may shift TNBC cells toward a transcriptional state associated with EMT inhibition and reduced aggressiveness. While the current study demonstrates the expression changes, future mechanistic work will be needed to determine whether these miRNAs directly mediate the functional effects of SMR peptide on TNBC behavior.

Three canonical tumor-suppressive miRNA axes appear central to the SMR response:

1. miR-200 → ZEB1/2 → EMT, restoring epithelial identity and reducing invasiveness [39,40].
2. miR-34a → p53/Notch/Wnt ± AXL/PI3K/AKT/Snail, constraining stemness and survival signaling [41].
3. let 7 → RAS/MYC ± HMGA2/LIN28, dampening proliferative drive [42].

Together, these axes provide a coherent mechanism for the SMR-mediated reduction of TNBC aggressiveness through miRNA-driven EMT reversal and down-tuning of oncogenic survival pathways [39,40,41,42,43].

**Figure 9:**
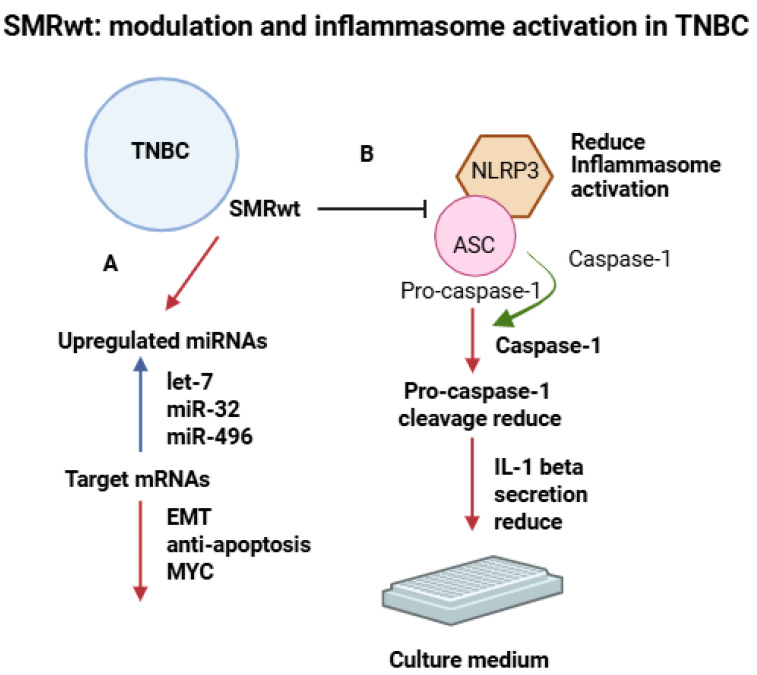
SMRwt-mediated modulation and inflammasome activation in TNBC. (**A**) Treatment of TNBC cells with SMRwt peptide leads to upregulation of specific microRNAs, including let-7, miR-32, and miR-496, which target mRNAs involved in EMT, anti-apoptotic pathways, and MYC signaling. (**B**) SMRwt also inhibits NLRP3 inflammasome activation by reducing ASC-mediated recruitment and cleavage of pro-caspase-1 into active caspase-1. This results in decreased IL-1β secretion into the culture medium, indicating suppression of inflammasome-driven inflammatory signaling. In the schematic, blue arrows denote SMRwt-induced upregulation of microRNAs, red arrows indicate downstream effects or reduced pathway outputs, green arrows represent enzymatic cleavage events, and blunt-ended black lines indicate inhibition. Created with BioRender.com.

Integation of the 11 miRNA panel (Figure 9) further supports broad SMR-mediated modulation of tumor-suppressive miRNAs—including let 7a/b, miR-26b-5p, miR-29a-3p, and miR-496 [44,45,46,47,48,49,50,51,52]—while also elevating several context-dependent/oncogenic miRNAs (miR-25-3p, miR-93-5p, miR-24-3p, miR-92a-3p, and miR-130a/b-3p) [53,54,55,56,57,58,59,60,61]. Despite these mixed shifts, the dominant network output remains anti-EMT and anti-survival, as reflected by downstream consequences—E cadherin gain, ZEB1/2 loss, reduced pAKT, and decreased Notch1 and nuclear β catenin [39,40,41,42,43,48,49,50,51,52,53,54,55,56,57,58].

At the pathway level, SMR-induced increases in miR-34a and miR-200 align with convergent suppression of PI3K/AKT and Wnt/β catenin, signaling axes tightly linked to EMT and therapeutic resistance [43]. These data collectively support a working model in which SMRwt enhances tumor-suppressive miRNAs → reduces EMT drivers and oncogenic pathways → limits TNBC plasticity, stemness, and survival.

Limitations include the need for causal validation through specific gain/loss miRNA perturbations, EMT phenotyping, pathway reporter assays, and temporal profiling across additional TNBC models to address context-dependent miRNA behavior.

From a translational standpoint, SMR appears to re-establish a tumor-suppressive miRNA network—involving miR-200, miR-34a, and let-7 axes—suggesting therapeutic potential in combination with AXL or PI3K inhibitors, miRNA-directed strategies, or nano-liposomal miRNA delivery approaches [62,63,64,65,66,67,68,69,70,71,72,73,74,75,76,77,78,79,80,81,82,83,84,85,86].

### 4.3. Integrated Pathway-Level Interpretation of SMR-Responsive miRNA Programs

The pathway enrichment patterns observed for the 11 SMR-associated miRNAs suggest that these regulators may converge on several signaling networks relevant to TNBC biology. Many of the enriched pathways, including ErbB and MAPK signaling, have established roles in breast cancer proliferation and survival [39]. Enrichment in endocytosis, focal adhesion, and ubiquitin-mediated proteolysis pathways aligns with known mechanisms of cytoskeletal remodeling, receptor turnover, and protein quality control that contribute to cancer progression [39]. The clustering of miRNAs such as miR-92a-3p, miR-128-15p, miR-25-3p, and miR-130/miR-148 family members suggests that these miRNAs may coordinate the regulation of multiple oncogenic and stress response pathways in parallel. While the results demonstrate significant pathway associations, additional functional studies will be necessary to determine how these miRNA–pathway interactions contribute to SMR-peptide-mediated effects in TNBC.

Pathway enrichment analysis of the top regulated miRNAs revealed that adhesion remodeling, vesicle trafficking, and proteostasis form a tightly interconnected stress-adaptation network that is prominently shaped by SMR-associated miRNA changes in TNBC cells. miRNAs enriched in focal adhesion and ECM-associated pathways point toward modulation of integrin signaling and cytoskeletal architecture, processes that directly influence TNBC cell motility and matrix engagement. Simultaneously, enrichment of endocytic and exocytic trafficking pathways suggests miRNA-level control over receptor turnover and vesicle secretion—mechanisms that determine the amplitude and duration of signals such as EGFR, integrins, and chemokine receptors, as well as the composition of secreted EVs. These structural and trafficking nodes converge with miRNA-regulated proteostasis modules involving ER stress responses, autophagy, and ubiquitin-mediated degradation, which together buffer proteotoxic stress and maintain signaling fidelity under hostile microenvironmental conditions. Notably, these functional axes feed into canonical proliferative circuits (e.g., MAPK and Hippo) that integrate mechanical cues, receptor dynamics, and metabolic stress to generate transcriptional outputs that control growth, survival, and invasion. Viewed as a whole, the clustering pattern suggests that SMR-induced miRNAs do not act in isolation but instead collectively reshape a conserved adaptive architecture that TNBC cells rely on to maintain plasticity and survive mechanical, metabolic, and proteotoxic challenges. By dampening adhesion-driven motility, altering receptor trafficking, and destabilizing proteostasis buffering, this miRNA program may shift TNBC cells toward a less invasive, less stress-resilient state—providing a mechanistic basis for the observed EMT suppression and offering potential therapeutic leverage points where peptide treatment and pathway-specific inhibitors could synergize to collapse compensatory survival circuits (Figure 4).

### 4.4. Network-Level Integration: miRNA Hubs, System-Level Control, and Therapeutic Implications

Across Figure 6, Figure 7 and Figure 8, the integrated miRNA–mRNA networks converge on a clear systems picture: a small set of highly connected miRNAs (e.g., let-7a/b-5p, miR-29a-3p, miR-24-3p, miR-30a-3p, miR-26b-5p, miR-92a/93-5p, miR-25-3p, miR-130a/b-3p, and the SMR-responsive miR-496) that co-target gene modules governing adhesion/ECM remodeling, vesicle trafficking, proteostasis, and proliferative signaling. These hubs provide a mechanistic link between modest miRNA shifts and coordinated pathway-level responses: they influence focal adhesion and endocytosis (receptor density and cytoskeletal organization), proteostasis pathways (ER stress, ubiquitin, and autophagy), and MAPK/Hippo signaling (growth and survival integration). This architecture reveals both robustness and actionable leverage points. Restoring tumor-suppressive hubs (let-7 and miR-29) while inhibiting pro-invasive hubs (miR-92/93/130) could redirect the stress adaptation network toward lower motility and reduced signaling output. The association of miR-496 with senescence pathways suggests that SMR-induced miRNA changes may also impose a senescence-like proliferative brake that complements EMT suppression. Therapeutically, these insights support two strategies: (i) hub-targeted miRNA modulation (let 7/miR-29 mimics; miR-92/93/130 inhibitors) and (ii) combinations pairing SMR treatment with pathway inhibitors aligned to hub-controlled nodes—such as FAK/integrin or EGFR/MEK blockade or agents disrupting autophagy and ER stress buffering. Because many hub signals appear in exosome-linked signatures, they also offer biomarker candidates for monitoring response and stratifying patients. Together, the network framework positions SMR-responsive hub miRNAs as central drivers of TNBC stress adaptation reprogramming and provides a translational roadmap for rational combination therapies.

The KEGG enrichment profiles for the 11 commonly upregulated miRNAs provide insight into potential regulatory networks influenced by SMR peptide treatment. Many enriched pathways, including focal adhesion, proteoglycans in cancer, MAPK signaling, and axon guidance—are known to play roles in cell migration, proliferation, and differentiation, processes central to TNBC progression [39]. The strong association of miR-130b-3p and miR-25-3p with focal adhesion and extracellular matrix-related pathways is consistent with previous reports linking these miRNAs to cytoskeletal remodeling and invasive behavior in cancer cells [39]. Likewise, enrichment of let-7 family miRNAs in MAPK signaling and axon guidance aligns with their documented functions in modulating proliferative signaling and maintaining cellular differentiation [39].

Recurrent enrichment of pathways related to protein processing in the endoplasmic reticulum, ubiquitin-mediated proteolysis, and cellular senescence suggests potential involvement in stress adaptation and proteostasis regulation—mechanistic themes previously proposed for SMR peptide activity in TNBC models [39]. Although the current analysis confirms pathway associations at the computational level, further experimental studies will be required to determine whether these miRNA-targeted pathways contribute directly to SMR-mediated phenotypic changes.

The coordinated upregulation of multiple miRNAs in MDA MB 231 cells following SMRwt exposure aligns with prior studies describing roles for these miRNAs in regulating proliferation, apoptosis, cytoskeletal dynamics, and metabolic adaptation in breast cancer models [39]. Increased expression of let 7 family members, miR-29a-3p, and miR-26b-5p is consistent with their documented tumor-suppressive roles, including repression of oncogenic transcription factors, modulation of extracellular matrix components, and attenuation of pro-metastatic signaling pathways [39]. In contrast, elevated levels of miR-92a-3p, miR-25-3p, miR-93-5p, and miR-130 family miRNAs may reflect compensatory regulation of signaling circuits associated with cell cycle control, DNA repair, and stress response pathways [39]. The observed induction of both tumor-suppressive and regulatory miRNAs supports the possibility that SMR peptide treatment influences broader transcriptomic programs relevant to TNBC cell behavior. Additional mechanistic studies will be necessary to determine whether the miRNA changes directly mediate phenotypic responses to SMR peptide.

### 4.5. SMRwt Peptide and the Inflammasome: Altered Inflammasome-Related Signaling in TNBC Cells

To test whether the SMRwt CPP peptide modulates inflammasome-related signaling through EVs, we evaluated EVs isolated from the below conditions: (1) untreated MDA MB 231 cells (231 UT EVs), (2) cells expressing the SMR mutant peptide (231 SMRmut EVs), (3) cells expressing the wild-type SMR cell-penetrating peptide (231 SMRwt EVs), and (4) SMRwt-expressing cells cultured under cholesterol-depleted conditions (SMRwt+CHO depletion EVs). This experiment was designed as a functional extension of our miRNA profiling results, which indicated that SMRwt-expressing TNBC cells selectively enrich pro-inflammatory and inflammasome-associated miRNAs within their EV cargo.

The SMRwt CPP peptide contains a defined cell-penetrating peptide (CPP) motif that enables rapid and efficient internalization into breast cancer cells, a mechanism extensively described in our earlier work [5,9,10]. Consistent with this established uptake pathway, naïve MDA MB 231 cells readily internalized SMRwt containing EV cargo following exposure.

Complementary transcriptomic profiling using the NanoString Breast Cancer 360 Panel demonstrated reduced expression of inflammasome-associated cytokines in TNBC cells relative to non-tumorigenic controls, including a log_2_ fold change of −1.15 for IL 1β (MDA MB 231 vs. MCF10A). 

In contrast, cholesterol-depleted SMRwt EVs failed to induce these responses and instead produced expression patterns comparable to the untreated control cells. This loss of activity was reflected in extracellular caspase 1 luminescence measurements (Figure 8), where SMRwt EVs produced a marked suppression of caspase 1 activity and cholesterol-depleted SMRwt EVs also did.

Because EV membrane structure, lipid raft organization, and cargo loading are cholesterol-dependent processes, these findings indicate that the pro-inflammatory signaling activity of SMRwt EVs—and their ability to modulate extracellular caspase 1—requires intact, cholesterol-rich EV membranes. Together, the data supports a model in which the SMRwt CPP peptide drives selective loading of immunomodulatory cargo into EVs in a cholesterol-dependent manner, enabling the delivery of pro-inflammatory signals to recipient TNBC cells.

As shown in Figure 8, MDA-MB-231 cells exposed to EVs derived from SMRwt-treated cultures exhibited a marked reduction in extracellular caspase-1 activity compared with EVs from untreated cells or SMRmut conditions. Cholesterol manipulation (via CHO treatment) partially restored caspase-1 activity in the presence of SMRwt, implicating membrane cholesterol/raft integrity in the SMRwt effect. EVs from the nonmalignant MCF10A line elicited significantly lower caspase-1 activity than MDA-MB-231 UT/SMRmut EVs. Together, these data indicate that SMRwt suppresses the robust release of active caspase-1 in MDA-MB-231 cells, likely via cholesterol-dependent membrane mechanisms that impact inflammasome signaling and/or EV cargo.

The caspase 1 data collectively suggest that SMRwt dampens inflammasome activation in TNBC cells, in contrast to both SMRmut and untreated tumor-derived EVs. This pattern is consistent with a broader theme emerging across the study: SMRwt disrupts key stress adaptation circuits in aggressive TNBC, not only at the level of miRNA hubs but also within innate immune signaling.

A reduction in EV-associated caspase 1 implies that SMRwt interferes with upstream steps of inflammasome engagement—such as NLRP3 assembly, ASC speck formation, or lipid raft dependent receptor priming. Because inflammasome activity contributes to chronic inflammation, pro-survival signaling, and pro-invasive remodeling in cancer cells, SMRwt-mediated suppression of caspase 1 is likely to shift TNBC cells toward a less inflammatory, less permissive state for invasion and stress tolerance.

Importantly, the partial reversibility of this effect by cholesterol manipulation supports the idea that SMRwt acts through membrane organization mechanisms, consistent with its known affinity for lipid raft or sphingomyelin-rich microdomains. This aligns with other SMR-associated phenotypes in the study—reduced EMT signatures, altered receptor trafficking, and suppression of pro-invasive miRNA hubs—all of which point to disruption of plasma membrane signaling architecture as a unifying mechanism.

The connection to the miRNA network is also notable: several SMR-responsive miRNAs (e.g., let-7 family, miR-29) have documented roles in modulating inflammatory pathways, NLRP3 signaling, or cytokine maturation. Thus, the inflammasome phenotype may reflect a combined effect of (i) SMRwt’s membrane level interference and (ii) the downstream rebalancing of miRNA hubs that control stress response genes. This provides a coherent systems model: SMRwt disrupts membrane organization → alters trafficking and receptor signaling → rewires miRNA regulated stress pathways → decreases inflammasome activation and inflammatory output.

The contrast between malignant and nonmalignant EV responses further reinforces this interpretation. TNBC-derived EVs appear intrinsically more capable of activating the inflammasome, whereas EVs from MCF10A cells do not. SMRwt reduces this tumor-specific hyperactivation, highlighting a potential therapeutic window in which SMRwt selectively targets cancer associated inflammasome signaling without broadly suppressing normal epithelial responses.

Overall, the inflammasome findings position SMRwt as a modulator of inflammatory and membrane-dependent stress programs in TNBC. Together with the miRNA-mediated network rewiring described above, these data support a model in which SMRwt attenuates pro-invasive, pro-survival inflammatory signaling and disrupts the ability of TNBC cells to maintain the membrane microdomains required for efficient inflammasome engagement. This integrated view strengthens the mechanistic foundation for developing SMR-based combination strategies aimed at disabling the compensatory circuits that sustain TNBC aggressiveness.

### 4.6. Integrating Mechanistic Insights of SMRwt Activity and Relevance to TNBC Therapeutic Gaps

To elucidate the molecular mechanisms underlying the anti-tumor activity of SMRwt in TNBC, we assessed its effects on miRNA regulation and inflammasome signaling—two pathways strongly implicated in TNBC progression but largely unaddressed by current therapies. Our findings reveal that SMRwt exerts coordinated effects on oncogenic transcriptional/post-transcriptional networks and inflammatory signaling, highlighting a dual mechanism of action highly relevant to persistent therapeutic challenges in TNBC. Taken together, these results may support a dual mechanism of SMRwt action: (i) reactivation of tumor-suppressive miRNA networks that inhibit EMT, MYC-driven transcriptional programs, and survival pathways [87,88,89,90,91] and (ii) inhibition of NLRP3 inflammasome signaling, thereby reducing IL 1β-mediated inflammation within the tumor microenvironment [92,93].

This combined targeting of intrinsic oncogenic pathways and extrinsic inflammatory drivers directly addresses two persistent therapeutic gaps in TNBC: the lack of effective molecularly targeted agents and the need to disrupt inflammation-driven immune evasion. By simultaneously modulating post-transcriptional regulatory hubs and dampening pro-tumorigenic inflammasome activity, SMRwt appears capable of destabilizing the compensatory stress adaptation circuits that underlie TNBC’s high plasticity, metastatic capacity, and treatment resistance.

Importantly, this integrated mechanism positions SMRwt as a multifunctional therapeutic strategy that impacts both tumor cell autonomous programs and the inflammatory cues within the tumor microenvironment that reinforce TNBC aggressiveness. The convergence of miRNA rewiring and inflammasome suppression suggests that SMRwt may reduce cellular adaptability while lowering inflammatory signaling thresholds that promote immune evasion and support metastatic dissemination.

Future studies should evaluate SMRwt in vivo TNBC models to determine whether these mechanistic effects translate into improved tumor control and reduce metastatic spread. Moreover, exploring potential synergy with standard chemotherapy or immune checkpoint inhibitors may reveal combination strategies that exploit SMRwt’s ability to disrupt both oncogenic and inflammation-driven survival pathways. Collectively, these insights nominate SMRwt as a promising therapeutic candidate targeting previously unaddressed biological vulnerabilities in TNBC.

### 4.7. Integrative Interpretation of Dysregulated miRNA Networks and Their Implications for TNBC Therapeutic Gaps

**Figure 10:**
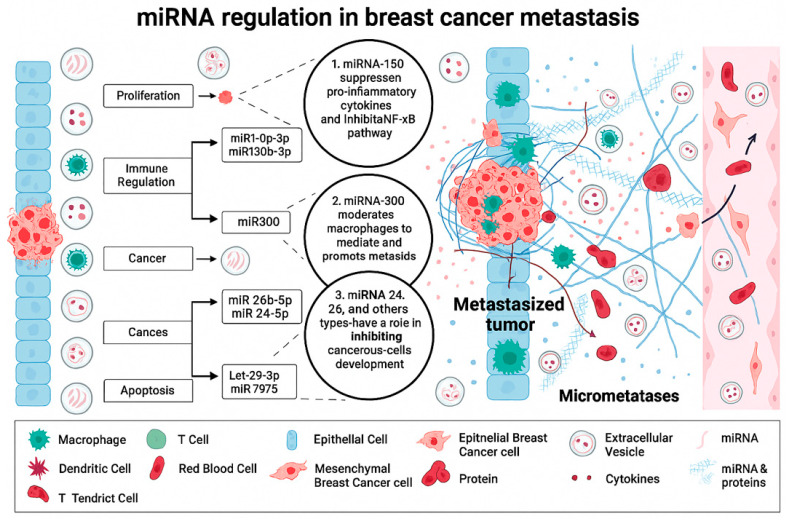
Role of EV-derived microRNAs in BC progression and metastasis. EVs secreted by epithelial BC cells transport miRNAs and proteins that regulate multiple processes during tumor progression. (Left) Distinct miRNAs modulate specific cellular pathways: 1. miR-130a-3p and miR-130b-3p promote proliferation by influencing pro-inflammatory cytokines and inhibiting NF-κB signaling. 2. miR-300 reprograms macrophages to support immune evasion and metastasis. 3. miR-26b-5p and miR-24-3p drive oncogenic transformation and cancer cell development. 4. Let-29a-3p and miR-7975 induce apoptosis by downregulating cell proliferation and survival pathways. These EV-mediated interactions facilitate metastatic dissemination, micrometastasis formation, and remodeling of the tumor microenvironment through dynamic crosstalk among cancer cells, immune cells (macrophages, dendritic cells, and T cells), cytokines, and circulating components such as red blood cells. Created with BioRender.com.

The integrative model presented in Figure 10 illustrates how dysregulated microRNA networks function across multiple cellular compartments to drive metastatic progression in breast cancer, emphasizing pathways highly relevant to triple-negative breast cancer (TNBC). The schematic highlights miRNA-mediated regulation in epithelial tumor cells, mesenchymal-like cancer cells, macrophages, and stromal elements, providing a system-level view of how disrupted post-transcriptional control shapes the tumor microenvironment (TME) and promotes micrometastatic niche formation. Because TNBC lacks approved targeted therapies and is driven by complex interactions among oncogenic signaling, inflammation, and stromal support, these insights underscore mechanistic gaps that miRNA-based interventions—such as those elicited by SMRwt—can help fill.

Members of the miR-130 family (miR-130a-3p and miR-130b-3p) are shown in the schematic as suppressors of pro-inflammatory signaling by inhibiting the NF-κB pathway. Chronic NF-κB activation is a hallmark of TNBC, sustaining survival, EMT, and chemotherapy resistance. Thus, the loss or downregulation of miR-130 removes an important brake on inflammatory cytokine production, contributing to the establishment of a TME that supports tumor expansion. Restoring miR-130 activity—either directly or indirectly through peptide-based modulation such as SMRwt—may therefore represent a strategy to attenuate NF-κB–driven inflammation, a long-recognized therapeutic vulnerability in TNBC.

In contrast, miR-300 is shown to promote macrophage-mediated tumor support, enhancing pro-tumorigenic polarization and facilitating metastatic dissemination. Macrophages play a particularly important role in TNBC progression, where tumor-associated macrophages (TAMs) foster immune evasion, survival of disseminated cells, and pre-metastatic niche conditioning. The depiction of miR-300 in the schematic emphasizes how miRNAs can regulate immune–tumor crosstalk and suggests that targeting miR-300 or its downstream pathways may disrupt macrophage support of early metastatic events—one of the key contributors to TNBC’s disproportionately high rate of recurrence and distant metastasis.

The inclusion of miR-24 and miR-26b-5p underscores their role in regulating cancer cell proliferation, differentiation, and survival. Dysregulation of these miRNAs contributes to the progression from localized breast cancer to an aggressive, invasive phenotype capable of intravasation and systemic dissemination. Their involvement across intersecting pathways underscores how small perturbations in miRNA networks can amplify oncogenic signals in TNBC, where cellular plasticity and adaptability complicate the efficacy of conventional therapies.

The schematic also illustrates the suppression of Let-7b-3p and miR-7975, both of which are associated with restricted migration and enhanced apoptosis. The Let-7 family is well established as a tumor suppressor frequently downregulated in TNBC, and its loss facilitates increased motility, stemness, and metastatic potential. Reduced Let-7b-3p expression, shown in the figure, aligns with the enhanced survival and migratory behavior necessary for generating micrometastases required for micrometastasis formation. Similarly, diminished miR-7975 activity promotes apoptotic resistance, further supporting metastatic survival under conditions of anchorage loss and immune surveillance.

Taken together, this integrative schematic emphasizes that metastatic progression in TNBC is not driven by a single dominant pathway but by a coordinated dysregulation of miRNAs that collectively modulate inflammation, immune programming, oncogenic proliferation, cell survival, and motility. These findings reinforce the notion that therapeutic approaches addressing only one node of TNBC biology are unlikely to be sufficient.

Importantly, the miRNA signatures depicted in Figure 10 converge on pathways dysregulated in TNBC that are not currently targeted by approved therapies, including: (i) chronic NF-κB–mediated inflammation, (ii) macrophage-derived pro-tumor support, (iii) MYC-driven transcriptional addiction, (iv) EMT-associated motility programs, and (v) evasion of apoptosis.

Agents such as SMRwt, which modulate multiple tumor-suppressive miRNAs while suppressing inflammatory signaling, align directly with these unmet needs. By restoring miRNA balance and attenuating inflammasome activity, SMRwt targets both intrinsic tumor biology and TME-driven metastatic programming—precisely the dual vulnerability highlighted in the schematic.

## 5. Conclusions and Future Directions 

SMRwt treatment induces broad molecular reprogramming in TNBC cells by modulating tumor-suppressive microRNAs and suppressing inflammasome activity. Profiling revealed that SMRwt upregulates key miRNAs—including let-7 family members, miR-26b-5p, miR-130a-3p, miR-32-3p, and miR-496—that are known to inhibit EMT, MYC-driven transcription, and anti-apoptotic signaling. Pathway enrichment and network analyses showed that these miRNAs target interconnected oncogenic pathways such as MAPK signaling, ubiquitin-mediated proteolysis, ER stress responses, and focal adhesion, suggesting coordinated disruption of TNBC survival mechanisms. Functionally, SMRwt significantly reduced NLRP3 inflammasome activation and IL 1β secretion in both cells and EVs, indicating attenuation of pro-inflammatory signaling. Together, these findings support a dual mechanism in which SMRwt restores miRNA-mediated tumor suppression and limits inflammasome-driven microenvironmental signaling. Future studies will focus on functional validation of key miRNAs, integrated multi-omics analysis, *in vivo* testing, mechanistic dissection of inflammasome inhibition, and optimization of therapeutic delivery.

## Data Availability

The data presented in this study is available on request from the corresponding author.

## Abbreviations

The following abbreviations are used in this manuscript.

BC	Breast Cancer
EVs	Extracellular Vesicles
SMR	Secretion Modification Region
EMT	Epithelial-to-Mesenchymal Transition
CHO	Cholesterol Manipulation
NLRP3	NOD-like receptor family pyrin domain-containing 3
ASC	Apoptosis-associated speck-like protein containing a CARD
RLU	Luminescence
UT	Untreated
WT	Wild Type
